# Supplementary figures and images for: GPT-4 generates accurate and readable patient education materials aligned with current oncological guidelines: A randomized assessment
Source: PLoS One. 2025 Jun 4;20(6):e0324175. doi: 10.1371/journal.pone.0324175 (PMC12136319; doi:10.1371/journal.pone.0324175)

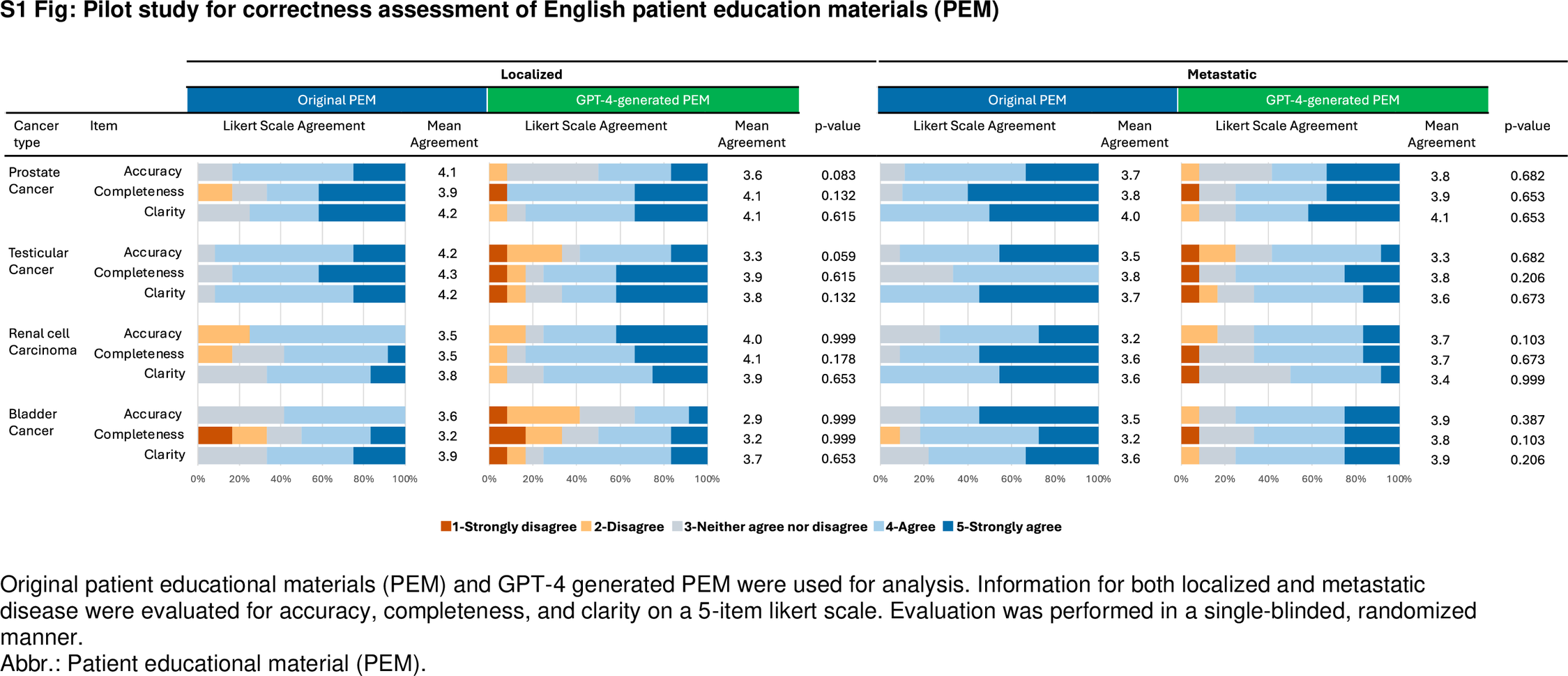

Supplement: S1 Fig — Original patient educational materials (PEM) and GPT-4 generated PEM were used for analysis. Information for both localized and metastatic disease were evaluated for accuracy, completeness, and clarity on a 5-item likert scale. Evaluation was performed in a single-blinded, randomized manner. Abbr.: Patient educational material (PEM). (TIF) [file pone.0324175.s001.tif]
