# Supplementary material for: GPT-4 generates accurate and readable patient education materials aligned with current oncological guidelines: A randomized assessment
Source: PLoS One. 2025 Jun 4;20(6):e0324175. doi: 10.1371/journal.pone.0324175 (PMC12136319; doi:10.1371/journal.pone.0324175)
Supplement: S2 Table — Used to translate the GenAI-generated PEMs into the respective language within the EU. Abbr.: Patient educational material (PEM). European Union (EU). (DOCX) [file pone.0324175.s003.docx]

**S2 Table: Prompt for PEM language translation**

| **Translation Prompt** |
| --- |
| Translate this [text] into (language: Dutch, German, French, Italian, Spanish):  Use a layperson language for patients that is understandable by or below a sixth-grade level, use a friendly and empathetic tone, keep the structure of the [text]. |

Used to translate the GenAI-generated PEMs into the respective language within the EU.

Abbr.: Patient educational material (PEM). European Union (EU).
