# Supplementary material for: GPT-4 generates accurate and readable patient education materials aligned with current oncological guidelines: A randomized assessment
Source: PLoS One. 2025 Jun 4;20(6):e0324175. doi: 10.1371/journal.pone.0324175 (PMC12136319; doi:10.1371/journal.pone.0324175)
Supplement: S3 Table — Availability of PEMs in spoken EU languages for prostate, kidney, bladder, and testicular cancer EAU treatment guidelines, based on October 10, 2023 query of the EAU patient portal (https://patients.uroweb.org). Abbr.: Patient educational material (PEM). European Association of Urology (EAU). (DOCX) [file pone.0324175.s004.docx]

**S3 Table:** Availability of original PEM

|  | **Prostate** | **Kidney** | **Bladder** | **Testis** | **Availability** |
| --- | --- | --- | --- | --- | --- |
| **English** | Yes | Yes | Yes | Yes | 100% |
| **German** | Yes | Yes | Yes | Yes | 100% |
| **Spanish** | Yes | Yes | No | No | 50% |
| **Dutch** | Yes | No | No | No | 25% |
| **French** | Yes | No | No | No | 25% |
| **Italian** | No | No | Yes | No | 25% |

Availability of PEMs in spoken EU languages for prostate, kidney, bladder, and testicular cancer EAU treatment guidelines, based on October 10, 2023 query of the EAU patient portal (<https://patients.uroweb.org>).

Abbr.: Patient educational material (PEM). European Association of Urology (EAU).
