# Supplementary material for: GPT-4 generates accurate and readable patient education materials aligned with current oncological guidelines: A randomized assessment
Source: PLoS One. 2025 Jun 4;20(6):e0324175. doi: 10.1371/journal.pone.0324175 (PMC12136319; doi:10.1371/journal.pone.0324175)
Supplement: S4 Table — Characteristics of study participants displayed based on YAU working group and language background. All native speakers are fluent in English and one of the five investigated EU-spoken languages. Abbr.: Young Academic Urologists (YAU). (DOCX) [file pone.0324175.s005.docx]

**S4 Table:** Baseline characteristics of raters assessing correctness of PEM

|  | **YAU Urotechnology Group**  (*n*=12) | **YAU Oncology Groups**  (*n*=20) | **Native speaker from YAU groups**  (*n*=10) |
| --- | --- | --- | --- |
| **Age**  Median  Range | 33  27-39 | 35  28-40 | 32.5  27-39 |
| **Gender**  Male  Female | 11  1 | 13  7 | 7  3 |
| **Training level**  Resident  Specialist | 2  10 | 5  15 | 3  7 |
| **YAU Member status**  Associate Member  Full member | 4  8 | 12  8 | 4  6 |

Characteristics of study participants displayed based on YAU working group and language background. All native speakers are fluent in English and one of the five investigated EU-spoken languages.

Abbr.: Young Academic Urologists (YAU).
