# Supplementary material for: GPT-4 generates accurate and readable patient education materials aligned with current oncological guidelines: A randomized assessment
Source: PLoS One. 2025 Jun 4;20(6):e0324175. doi: 10.1371/journal.pone.0324175 (PMC12136319; doi:10.1371/journal.pone.0324175)
Supplement: Attachment — Submitted filename: PEM_ReviewResponse1_03292025.docx [file pone.0324175.s008.docx]

**Journal Requirements And Editor Comments**

**Thank you for submitting your manuscript "Guideline-based patient educational materials using generative artificial intelligence" to PLOS ONE. Please accept our sincere apologies for the delay in getting this report to you. The difficulty in securing appropriate reviewers with expertise in both urological education materials and artificial intelligence applications resulted in a longer review process than anticipated.
We have now received comments from one reviewer, and I have also personally reviewed the manuscript in detail - See this under editor comments below. Both reviews identify several strengths in your work, particularly the novel application of GenAI to creating patient educational materials with improved readability while maintaining comparable accuracy to traditional methods.
However, there are several methodological issues and areas for improvement that need to be addressed before your manuscript can be considered further. I have attached both the reviewer's comments and my own editorial assessment for your consideration.**

*Response:* We thank the editor for the diligence in finding sufficient reviewers for this manuscript given the expertise of the reviewers required, and we understand the delay as part of the publishing process. We are deeply grateful to the editor for providing a review of the manuscript, ensuring it undergoes a thorough review and is made more robust in exploring the role of GenAI in patient education materials (PEM). We appreciate the reviewers’ comments regarding the novel utility of GenAI in this space and will address the methodological and other concerns in the responses below.

**1) Please ensure that your manuscript meets PLOS ONE's style requirements, including those for file naming.**

*Response:* We have modified the manuscript to accurately reflect the PLOS ONE style requirements, ensuring proper formatting and file naming.

**2. Please note that PLOS ONE has specific guidelines on code sharing for submissions in which author-generated code underpins the findings in the manuscript. In these cases, we expect all author-generated code to be made available without restrictions upon publication of the work. Please review our guidelines at** [**https://journals.plos.org/plosone/s/materials-and-software-sharing#loc-sharing-code**](https://urldefense.com/v3/__https:/journals.plos.org/plosone/s/materials-and-software-sharing*loc-sharing-code__;Iw!!JkGBRS3n8cDS!jlvTZobX5m_oIpD_96Ab813h10YEEGydB7dn8uNvwORVRBhgxq8bpWzB0CT1cB2IdfR64AEdhbiZP5Qq0VVMU3-2bCo$) **and ensure that your code is shared in a way that follows best practice and facilitates reproducibility and reuse.**

*Response:* No code was used in this study.

**3. Thank you for stating the following in your Competing Interests section:**

**“NONE”**

**Please complete your Competing Interests on the online submission form to state any Competing Interests. If you have no competing interests, please state "The authors have declared that no competing interests exist.", as detailed online in our guide for authors at** [**http://journals.plos.org/plosone/s/submit-now**](https://urldefense.com/v3/__http:/journals.plos.org/plosone/s/submit-now__;!!JkGBRS3n8cDS!jlvTZobX5m_oIpD_96Ab813h10YEEGydB7dn8uNvwORVRBhgxq8bpWzB0CT1cB2IdfR64AEdhbiZP5Qq0VVMCyH0hpc$)

**This information should be included in your cover letter; we will change the online submission form on your behalf.**

*Response:* We have modified the cover letter to properly include recently developed conflicts of interest and will include the statement in the online submission.

**4. In the online submission form you indicate that your data is not available for proprietary reasons and have provided a contact point for accessing this data. Please note that your current contact point is a co-author on this manuscript. According to our Data Policy, the contact point must not be an author on the manuscript and must be an institutional contact, ideally not an individual. Please revise your data statement to a non-author institutional point of contact, such as a data access or ethics committee, and send this to us via return email. Please also include contact information for the third party organization, and please include the full citation of where the data can be found.**

*Response:* We thank the editor for the comment. Please consider that the initial page of the manuscript was included for internal purposes and is not applicable for the submission of the manuscript. We have eliminated this page as it does not comply with the PLOS guidelines for submission. Further, we have included the raw data in the submission.

**5. Please include a separate caption for each figure in your manuscript.**

*Response:* Captions have been appropriately included for all main figures/tables in the manuscript.

**6. Please include captions for your Supporting Information files at the end of your manuscript, and update any in-text citations to match accordingly. Please see our Supporting Information guidelines for more information:** [**http://journals.plos.org/plosone/s/supporting-information**](https://urldefense.com/v3/__http:/journals.plos.org/plosone/s/supporting-information__;!!JkGBRS3n8cDS!jlvTZobX5m_oIpD_96Ab813h10YEEGydB7dn8uNvwORVRBhgxq8bpWzB0CT1cB2IdfR64AEdhbiZP5Qq0VVMgspHBTY$)**.**

*Response:* Captions have been appropriately added for all supplemental figures/tables.

**Please review your reference list to ensure that it is complete and correct. If you have cited papers that have been retracted, please include the rationale for doing so in the manuscript text, or remove these references and replace them with relevant current references. Any changes to the reference list should be mentioned in the rebuttal letter that accompanies your revised manuscript. If you need to cite a retracted article, indicate the article’s retracted status in the References list and also include a citation and full reference for the retraction notice.**

*Response:* Citations have been reviewed to ensure accuracy and completeness.

**Additional Editor Comments**

***Abstract:***

**1) Line 184, page 4, under abstract – the patient summary could be slightly expanded to match the level of detail in the rest of the abstract while maintaining its improved readability. Also, ‘patient info,’ should be written in full.**

*Response:* We thank the editor for the comment and have expanded the patient summary as follows:

*“Some cancer facts made for sick people can be hard to read or not in the right words for those with prostate, bladder, kidney, or testicular cancer. This study used AI to quickly make short and easy-to-read content from trusted facts. Doctors checked the AI content and found that they were just as accurate, complete, and clear as the original text made for patients. They also worked well in many languages. This AI tool can assist providers in making it easier for patients to understand their cancer and the best care they can get.”* (Page 3, lines 129-135)

**2) Line 161, page 4 – Young academic urologist (YAU).. revise to ensure consistency in capitalisation.**

*Response:* We thank the editor for the correction and have revised YAU as “Young Academic Urologists” across all instances.

***Introduction:***

**1) Consider starting with the challenge of health literacy and readable PEMs before mentioning internet statistics.**

*Response:* We thank the editor for the comment and agree that health literacy is a major challenge and should be featured at the start of the introduction. We have modified the initial paragraph to include the sentences as follows:

*“Health literacy remains an often-overlooked factor in health disparities, with research directly linking low literacy to negative health outcomes[1, 2]. This is in part due to the dearth of validated medical information that is written specifically for patients, forcing many of them to turn to less reliable sources on the internet and potentially compromise care[3]. Approximately 4.5% of all internet search queries are health-related, totaling around 6.75 million health-related searches each day[4]. Given this, countering the exposure to potential misinformation through the creation of readable, correct, and up-to-date patient educational materials (PEMs) is a fundamental objective of societies across specialties[5].”* (Page 4, lines 147-154)

Studies cited as follows:

N. D. Berkman, S. L. Sheridan, K. E. Donahue, D. J. Halpern, and K. Crotty, "Low health literacy and health outcomes: an updated systematic review," (in eng), Ann Intern Med, vol. 155, no. 2, pp. 97-107, Jul 19 2011, doi: 10.7326/0003-4819-155-2-201107190-00005.

[G. Bartlett, R. Blais, R. Tamblyn, R. J. Clermont, and B. MacGibbon, "Impact of patient communication problems on the risk of preventable adverse events in acute care settings," (in eng), Cmaj, vol. 178, no. 12, pp. 1555-62, Jun 3 2008, doi: 10.1503/cmaj.070690.

S. Loeb et al., "Fake News: Spread of Misinformation about Urological Conditions on Social Media," (in eng), Eur Urol Focus, vol. 6, no. 3, pp. 437-439, May 15 2020, doi: 10.1016/j.euf.2019.11.011.

G. Eysenbach and C. Kohler, "What is the prevalence of health-related searches on the World Wide Web? Qualitative and quantitative analysis of search engine queries on the internet," (in eng), AMIA Annu Symp Proc, vol. 2003, pp. 225-9, 2003.

B. Weis, "Health Literacy: A Manual for Clinicians. Chicago, IL: American Medical Association, American Medical Foundation; 2003.• National Institutes of Health. How to Write Easy to Read Health Materials: National Library of Medicine Website," How to Write Easy to Read Health Materials: National Library of Medicine Website.

**2) A brief mention of previous research, specifically on AI in medical education materials, would strengthen your argument.**

*Response:* We agree with the editor’s comment and have included a sentence with two cited studies, one in ENT and one in urology, which have used generative-AI tools for the specific goal of simplifying patient education materials. This demonstrates the potential for improved PEMs using GenAI as discussed in the literature.

The sentence added is as follows:

*“Previous studies have shown that GenAI tools like ChatGPT can be used to simplify patient education materials in multiple specialties with comparable correctness and improved readability.”* (Page 4, lines 172-174)

Citations are as follows:

Y. B. Shah, A. Ghosh, A. Hochberg, J. R. Mark, C. D. Lallas, and M. S. Shah, "Artificial intelligence improves urologic oncology patient education and counseling," (in eng), Can J Urol, vol. 31, no. 5, pp. 12013-12018, Oct 2024.

E. A. Patel et al., "The Use of Artificial Intelligence to Improve Readability of Otolaryngology Patient Education Materials," (in eng), Otolaryngol Head Neck Surg, vol. 171, no. 2, pp. 603-608, Aug 2024, doi: 10.1002/ohn.816.

**3) Add a sentence addressing the potential ethical implications or limitations of GenAI in medical information.**

*Response:* We thank the editor for the comment regarding the important discussion of ethical implications and limitations with this technology. Regarding the limitations, these are briefly discussed in the introduction citing the study by *Davis et al.* which identified limitations in *“consistent correctness when relying on the proprietary knowledge domain*”. We feel this inclusion is a sufficient introduction to the limitations, however we will expand on the limitations of GenAI in the discussion section. Regarding the ethical implications, we have included a reference to *Eppler et al.,* which conducted a global survey of urologists on the use of GenAI. The study found that while urologists are incorporating GenAI in patient education, they remain concerned about ethical issues including the risk of bias and patient privacy. We have added a sentence exploring these ethical issues as follows:

*“However, a global survey of urologists found that while GenAI is useful for patient education, there are ethical concerns regarding the potential spread of biased information and patient privacy breaches[12].”* (Page 4, lines 167-169)

Citation is as follows:

M. Eppler et al., "Awareness and Use of ChatGPT and Large Language Models: A Prospective Cross-sectional Global Survey in Urology," (in eng), Eur Urol, vol. 85, no. 2, pp. 146-153, Feb 2024, doi: 10.1016/j.eururo.2023.10.014.

R. Davis et al., "Evaluating the Effectiveness of Artificial Intelligence-powered Large Language Models Application in Disseminating Appropriate and Readable Health Information in Urology," (in eng), J Urol, vol. 210, no. 4, pp. 688-694, Oct 2023, doi: 10.1097/ju.0000000000003615.

**4) Consider including a line about the novelty/gap your study addresses**

*Response:* We thank the reviewer for the comment and have added a sentence elucidating the novelty of the study, which is primarily in the use of guidelines as the data source for extraction using custom GenAI model to improve the accuracy of PEMs. Another novelty is the translation of the PEMs into various EU languages to fill the gaps in current PEMs which are written only in English and German for all four urologic cancers. The sentences added and modified are as follows:

*“In this study we assessed the potential of GenAI to extract guideline information from the European Association of Urology (EAU), simplify it for better patient understanding, and translate it into multiple languages - bridging the accessibility gap for certified healthcare content."* (Page 4, lines 170-172)

*“While these studies relied on the base GenAI model, our study represents a novel approach through the creation of a custom model to extract information from recent urologic oncology guidelines, mimicking the standard workflow for PEM development.”* (Page 4, lines 174-177)

**5) Line 206: "on yearly bases" should be "on a yearly basis"**

**Line 214: "different scholarity levels" - Consider using "educational" or "literacy" levels instead of "scholarity"**

**Line 211: "but lacks consistent correctness when relying on the proprietary knowledge domain" - This phrase is somewhat unclear; consider rewording for clarity**

**Line 209: Consider rephrasing "not meeting requirements set by the European Union (EU) for understandability of clinical summaries" for better clarity**

*Response:* We thank the editor for their suggested improvements and have incorporated them into the introduction for increased clarity.

***Methods:***

**1) The "GPT-4 framework development and custom-GPT creation" mentioned in the opening paragraph is never fully explained. Please provide some explanation to complement the figure provided to ensure reproducibility.**

*Response:* We thank the editor for the comment. In the section on “GenAI Framework Development”, we discuss the development of the tri-phasic framework which builds off the prompt in *Eppler et al.* and can be found in the supplementary materials of that study. The framework in *Eppler et al.* was designed as a 2-layer system to extract relevant information from text and summarize it at a particular reading level. The tri-phasic framework as outlined in our methods “was utilized to extract relevant information from clinical guidelines (phase 1) and adapt it to the readability level recommended by the European Union (phase 2), as well as to translate the English output into languages commonly spoken within the EU (Phase 3).” This framework was distilled into a prompt and used to build a custom GPT that has been linked in the methods section <https://chatgpt.com/g/g-sZZBNfGSl-patient-education-material-readible-summaries>. The translation prompt has been included the supplementary materials (S2 Table). To remove any confusion and discrepancy with Figure 1, the key portions of the triphasic framework are now highlighted in the Figure 1 caption as follows:

*“Figure 1. Pipeline for generating layperson summaries of medical trials using a tri-phasic large language model (LLM)-based framework with human oversight. The process starts with input from the most current guidelines, which the LLM processes in stages. First, key sections (localized disease, metastatic disease and treatments of each section) are identified (phase 1) and simplified into lay terms (phase 2). The output is then translated into five languages commonly spoken in the EU (phase 3).”*

**2) Regarding your prompting methodology for GPT-4, while you mention that "prompts were based on previously published methods and modified to extract guideline information," this provides insufficient detail for reproducibility. Please consider enhancing this section by:**

- **Including at least one complete example prompt**
- **Specifying the exact modifications made to previously published methods**
- **Detailing any GPT-4 parameters used (temperature, token limits, etc.)**
- **Explaining how your prompts specifically targeted EU-recommended reading levels**
- **Describing any iterative refinement process used to optimize the prompt.**

*Response:* We thank the editor for their comment regarding prompting methodology and agree that clarification of the methodology is essential for reproducibility. We believe that our inclusion that *“The prompts used were based on previously published methods[11] and were modified to extract key guideline information and generate an overview of therapeutic recommendations for both localized and metastatic disease, written to compile with the EU-recommended patient reading level.”* (Page 5, lines 198-200) is mostly sufficient as it describes the modifications made to the previously published prompt which is accessible in the cited paper by *Eppler et al.* Additionally, the custom GPT is provided in the manuscript (<https://chatgpt.com/g/g-sZZBNfGSl-patient-education-material-readible-summaries>), which should be used for the reproducibility of findings as it is an exact replication of the GenAI-generation protocol used in this study. As there is no custom GPT available for the translation component of the tri-phasic framework, we have included the prompt in the supplementary materials (S2 Table). To add some clarity, we have added and modified the sentences as follows:

*“The prompts were refined until a consistent output in three consecutive tests was achieved. Temperature and token limits were not adjusted from the base GPT-4 model.”* (Page 5, lines 200-202)

*“A custom GPT, utilizing the same prompt for generating PEMs, was developed for free use and to validate the findings of this study:* [*https://chatgpt.com/g/g-sZZBNfGSl-patient-education-material-readible-summaries*](https://chatgpt.com/g/g-sZZBNfGSl-patient-education-material-readible-summaries)*. The prompt for translating the GenAI-generated PEMs can be found in S2 Figure.”* (Page 6, lines 207-210)

Studies cited:

M. B. Eppler et al., "Bridging the Gap Between Urological Research and Patient Understanding: The Role of Large Language Models in Automated Generation of Layperson's Summaries," (in eng), Urol Pract, vol. 10, no. 5, pp. 436-443, Sep 2023, doi: 10.1097/upj.0000000000000428.

**3) Regarding the sample size of reviewers (12 for pilot, 20 for main assessment with 5 per cancer type), I note the absence of a formal justification for these numbers. While traditional power calculations may be challenging for expert review studies, I recommend:**

- **Providing a brief rationale for the chosen sample sizes based on:**
  - **Previous similar validation studies in medical education/PEM literature**
  - **Practical considerations regarding YAU expert availability**
  - **Anticipated effect sizes based on pilot results**
- **Acknowledging this limitation and discussing how you addressed potential reliability concerns with a relatively small number of reviewers per cancer type**

*Response:* We thank the editor for the comment regarding the sample size of reviewers. We agree that there should be a formal justification for these numbers included in the text and appreciate the detailed suggestions on how to ensure proper justification. Studies (cited below Zamanzadeh et al., E. Fernández-Gómez et al.) have shown that there is no consensus on the number of experts required for the validation of patient education materials and that it can range from 2 to dozens of reviewers. Another study determined that 5 reviewers is sufficient to control chance agreement. Previous studies looking specifically at GenAI in patient education utilized 2 (Shah) and 3 (Patel) independent reviewers respectively. Regarding YAU expert availability, each working group is made up of between 23-35 individuals, many of which lack native English fluency or were occupied with other tasks. Given this, the chairs of each working group assigned 5 members to the task who were best equipped for the randomized assessment. Our resulting expert sample represents 14%, 19%, 17%, and 22% of prostate, bladder, kidney, and testis cancer working groups respectively. Regarding the pilot results, the pilot study with 12 urologists from the YAU group for urotechnology revealed no differences in the ratings for all three outcomes accuracy, completeness and clarity (S1 Fig). Effect sizes were not considered given the limited availability of YAU working group members for the randomized assessment. We will acknowledge the number of reviewers as a potential limitation, with the caveat that any potential significance gained from additional reviewers would have limited meaningfulness given that small differences detected with increased power would have limited practical utility. To add clarity, the manuscript has been edited as follows:

*“Research indicates a lack of consensus on the optimal number of expert evaluators needed, with findings suggesting that 5 reviewers are sufficient to control chance agreement [23, 24]. Experts from each YAU working group were selected by the respective YAU chairs as best suited to evaluate PEMs. Our resulting expert panel represents 14%, 19%, 17%, and 22% of prostate, urothelial, kidney, and testis cancer working groups respectively.”* (Page 8, lines 277-281)

*“Several limitations must be considered. First, the number of expert reviewers in the randomized assessment was limited due to the availability of YAU working group members.”* (Page 13, lines 434-435)

The studies cited are as follows:

V. Zamanzadeh, A. Ghahramanian, M. Rassouli, A. Abbaszadeh, H. Alavi-Majd, and A. R. Nikanfar, "Design and Implementation Content Validity Study: Development of an instrument for measuring Patient-Centered Communication," (in eng), J Caring Sci, vol. 4, no. 2, pp. 165-78, Jun 2015, doi: 10.15171/jcs.2015.017.

E. Fernández-Gómez, A. Martín-Salvador, T. Luque-Vara, M. A. Sánchez-Ojeda, S. Navarro-Prado, and C. Enrique-Mirón, "Content Validation through Expert Judgement of an Instrument on the Nutritional Knowledge, Beliefs, and Habits of Pregnant Women," (in eng), Nutrients, vol. 12, no. 4, Apr 18 2020, doi: 10.3390/nu12041136.

**4) There is no mention of translation validation methodology or back-translation verification.**

*Response:* We thank the editor for the comment regarding the methodological framework for evaluating PEM translations. Regarding back-translation, we chose not to conduct a back-translation for two reasons. First, there has been significant work in recent years demonstrating the flaws of back-translation as a reliable validation tool, particularly in bilingual settings due to an over-reliance on exact equivalence without incorporating cultural or linguistic context (Colina et al. 2017, Douglas & Craig 2007). Second, given the stochasticity and context-dependent formulation of GenAI, it is not designed to produce exact word-for-word translations but may in fact produce more clear and cohesive translations which could potentially be penalized unfairly by back-translation. Rather we favored an expert team approach to assess the translations. As there are currently no widely accepted translation validation tools, previous studies have designed their own metrics to assess quality of GenAI-generated translations (Gulati et al. 2024). Given this context, we utilized a modified expert team approach focused on the correctness and clarity of the translation. To make this clearer in the text and avoid confusion regarding “correctness” in translation vs “correctness trifecta” for layperson simplification, we have changed the terminology from “correctness” to “faithful” translation throughout the manuscript while maintaining the same definition used in the survey. Further, we have modified the methodology as follows:

*“Translations to Dutch, French, German, Italian and Spanish were assessed for faithfulness and clarity on a 5-item Likert scale (1=strongly disagree, 2=disagree, 3= neither agree nor disagree, 4=agree, 5=strongly agree). Faithfulness was defined as the level of accuracy and completeness compared to the English PEM and clarity was defined as the level of coherence as described above. An expert team approach to language translation was used for independent evaluation with two bilingual members originating from different YAU groups who speak the respective language at a native fluency[25].”* (Pages 8-9, lines 292-298)

Studies cited:

Colina, S., Marrone, N., Ingram, M., & Sánchez, D. (2017, September). Translation Quality Assessment in health research: A Functionalist alternative to back-translation. Evaluation & the health professions. <https://pmc.ncbi.nlm.nih.gov/articles/PMC6800023/>

Douglas, S., & Craig, S. (2007). Collaborative and iterative translation: An alternative approach to back translation. <https://journals.sagepub.com/doi/10.1509/jimk.15.1.030>

Gulati V; Roy SG; Moawad A; Garcia D; Babu A; Poot JD; Teytelboym OM. 2024. Transcending language barriers: Can chatgpt be the key to enhancing multilingual accessibility in health care?. Journal of the American College of Radiology : JACR. <https://pubmed.ncbi.nlm.nih.gov/38880289/>

**5) No explanation of how specific PEMs were selected for evaluation or whether they represent the full spectrum of urological cancers.**

*Response:* We thank the editor for the comment. In the section on “Data sources” we identify the EAU website and state that “*We selected the information for prostate cancer, bladder cancer, renal cell carcinoma, and testicular cancer management.*” (Page 6, lines 216-217) In this case, we mention that we focused on “*first-line treatment for metastatic disease, as patients most often search the internet for information regarding disease treatment specifically[16-20].”* (Page 6, lines 229-231) Regarding the spectrum of urologic cancers, prostate, bladder, renal cell carcinoma (RCC), and testis cancer make up the 4 most common urologic cancers. Other rare cancers including adrenal cancer and penile cancer exist but have very low prevalence in the population and are not frequently seen in urologic practice. To add clarity to the cancers represented, we have modified the methods as follows:

*“We selected the information for the management of common urologic cancers - prostate cancer, bladder cancer, renal cell carcinoma, and testicular cancer.”* (Page 6, lines 216-217)

**Results:**

**1) Line 356: Mentions "translations to Dutch, French, German, Italian and French" - French is listed twice, likely a typo**

**Line 386: p-value written as "p=738." instead of "p=.738"**

*Response:* We thank the editor for identifying these inaccuracies and they have been corrected in the manuscript text.

**2) Line 372-373: Only reports median values (4) for all measures in the pilot study, making it impossible to detect nuanced differences**

*Response:* We thank the editor for the comment and agree that the reported median might not fully detect nuanced differences despite its common use with ordinal variables. As requested, we changed all values to mean and standard deviation as already performed in the section “Readability assessment of English PEMs” within the results. Therefore, the entire results section has been rewritten and recalculated. Additionally, relevant figures and supplementary materials have been updated accordingly. While not having impact on the calculated p-values, minor differences in mean values can now be detected.

**33) The study reports numerous non-significant p-values but no effect sizes or confidence intervals.**

*Response:* We thank the editor for identifying this weakness in the calculations presented in the first version of this manuscript. As we have already reported on means and standard deviation in the first section of the results, we have now adapted the entire manuscript and have recalculated the remaining sections. Through the displayed mean values, nuanced differences in ratings can be detected. The standard deviation provides a better understanding of the potential variance in the ratings. Figure 3, figure 4 and supplementary figure 1 have been adopted to this request as well to provide consistency throughout the entire manuscript.

**44) No interrater reliability metrics (e.g., Fleiss' kappa) are reported despite multiple raters.**

*Response:* We thank the editor for identifying this omission. We have now calculated the interrater reliability for both cohorts and have included the values in the results section as follows:

*“Interrater reliability was calculated by Fleiss' kappa.”* (Page 9, lines 307-308)

*“In this pilot experiment, the mean (SD) accuracy [original: 3.6 (0.8) vs GPT-4: 3.6 (1.1)] , completeness [original: 3.6 (1.1) vs GPT-4: 3.8 (1.1)], and clarity [original: 3.9 (0.8) vs GPT-4: 3.8 (1.0)] for original PEM as well as GPT-4 generated PEM was not significant different for each scenario with an Fleiss' kappa of -0.027 (p=.062, S1 Fig).“* (Page 10, lines 330-333)

*“Afterwards, twenty urologists with a proven track record in the respective field of uro-oncology and blinded for origin of the PEM rated the accuracy, completeness, and clarity in a randomized way (Fleiss' kappa: 0.007, p=.460)”* (Page 10, lines 335-337)

**5) Results show identical median values (mostly 4) for almost all comparisons, raising questions about the sensitivity of your measurement scale.**

*Response:* We appreciate the editor’s comment regarding the median values and acknowledge this as a limitation seen in all studies utilizing a Likert scale for evaluating GenAI-generated content. To rectify this, we have presented the data using means and standard deviations, giving us a marginal greater ability to detect nuanced differences. Unfortunately, at the time of submission, no validated tools exist for assessing the correctness of GenAI-generated content, making Likert scales the most widely used method for evaluating output quality. We have addressed this limitation in the manuscript as follows:

*“Further, while Likert scales are often used in the evaluation of GenAI-generated content[11, 13], the ability to detect differences is often limited given the clustering of responses. Validated tools for evaluating the quality of GenAI-generated content are needed.”* (Page 13, lines 438-440)

**Discussion:**

**1) No discussion of why the readability metrics were better for GenAI but accuracy and clarity ratings were similar.**

*Response:* We thank the editor for raising an important point regarding the differences between readability and quality metrics. Readability metrics and grade level indicators are designed to assess and quantify sentence complexity using various criteria including sentence length and syllable count (Badarudeen 2010). These formulas tie sentence criteria to grade levels and reading levels through validation against comprehension studies. While readability metrics serve as potential proxies for understandability, the quality of the text is not fully addressed. PEMs may be readable but lack accuracy and clarity if they misrepresent the original guidelines or include syntactical errors that are not measured by readability metrics. Given this, human supervision and editing remains a key step to ensure that GenAI PEMs maintain their quality. To underscore this point, we have added a discussion as follows:

*“While GenAI-generated PEMs showed improved readability, they achieved similar levels of correctness and quality in representing the guidelines as the original PEMs. Given that readability metrics do not consider the quality of the writing, optimizing for readability is not sufficient for ensuring proper dissemination of PEMs[32]. Resolving the limitations may be possible through integration of GenAI into the workflow of updating PEMs. Integration with human verification and editing might allow organizations to keep up with the workload of guideline changes and increase readability while also maintaining the quality (Fig 1).”* (Page 12, lines 400-405)

The studies cited are as follows:

S. Badarudeen and S. Sabharwal, "Assessing readability of patient education materials: current role in orthopaedics," (in eng), Clin Orthop Relat Res, vol. 468, no. 10, pp. 2572-80, Oct 2010, doi: 10.1007/s11999-010-1380-y.

**2) Limited exploration of the variability in translation quality (simply stating "majority of cases" is imprecise).**

*Response:* We thank the reviewer for the comment and acknowledge the quality of the translation needs to be explored further. As discussed above, we replaced “correct” with “faithful” for more precise language and to avoid confusion with the correctness trifecta. We further explored the variability and acknowledged the limitations of the GenAI models in translating medical information; the technology cannot stand alone as the sole translator given the lower quality scores in one-third of cases. While the translation capabilities will likely improve with future GenAI models, native-speaking urologists and certified translators should always work to finalize the output – enhancing quality while maintaining readability. The manuscript has been updated as follows:

*“Translations of GenAI-generated PEMs were rated as clear and faithful in more than two-thirds of cases, enabling the potential dissemination within the member states of the EAU after further refinement.”* (Page 11, lines 364-366)

*“This same workflow extends to translating PEMs. While the language capabilities will likely improve with future models[33], native-speaking urologists and certified translators should always work to finalize the translations.”* (Page 12, lines 405-407)

The studies cited are as follows:

H. Nori *et al.*, "Can Generalist Foundation Models Outcompete Special-Purpose Tuning? Case Study in Medicine," *arXiv preprint arXiv:2311.16452,* 2023.

**3) No explanation for the seemingly contradictory finding that GenAI PEMs were less favored for localized disease but more favored for metastatic disease (line 421-423)**

*Response:* We thank the reviewer for the comment. Regarding the finding on GenAI PEMs favored for metastatic disease, that could be in part due to the rapid changes that are occurring with chemotherapy development not captured by original PEMs due to infrequent updating. We discussed this topic in the discussion: *“Original PEMs might not be updated regularly. For instance, the information on bladder cancer for metastatic disease does not cover novel developments regarding immunotherapy and combination therapies with antibody drug conjugates such as enfortumab vedotin, despite entering routine care”*. (Page 12, lines 388-391) To make our hypothesis more explicit, we have added the sentence as follows:

*“This may contribute to the favoring of GenAI-generated PEMs which can be more easily updated in tandem with guideline releases.”* (Page 12, lines 391-392)

**4) Limited connection to broader AI ethics literature or frameworks for medical AI implementation.**

**No discussion of regulatory considerations for AI-generated patient materials.**

*Response:* We thank the editor for pointing out the essential inclusion of AI ethics literature and regulatory frameworks when discussing GenAI-generated PEMs. To add to the discussion, we added a short paragraph discussing the ethical AI initiatives currently taking place in academic research and recent the AI Act of 2024 passed by the EU to regulate the use of AI in medicine. These efforts crucial aspects of the AI ethics and regulation conversation pertaining to PEMs. The section has been added as follows:

*“Regarding ethical practice, initiatives are underway to ensure the ethical and responsible use of GenAI as an intervention in academic research[40]—an essential step for piloting patient-facing tools. Similar methods have been spearheaded by the EU through the AI Act of 2024, which classifies patient-facing GenAI models as “low risk” and requires proper disclosure with oversight by individuals with sufficient “AI literacy”[41].”* (Page 13, lines 421-425)

The studies cited are as follows:

G. E. Cacciamani, G. S. Collins, and I. S. Gill, "ChatGPT: standard reporting guidelines for responsible use," (in eng), Nature, vol. 618, no. 7964, p. 238, Jun 2023, doi: 10.1038/d41586-023-01853-w.

H. van Kolfschooten and J. van Oirschot, "The EU Artificial Intelligence Act (2024): Implications for healthcare," Health Policy, vol. 149, p. 105152, 2024/11/01/ 2024, doi: https://doi.org/10.1016/j.healthpol.2024.105152.

**5) Minimal connection to patient communication theory or health literacy research.**

*Response:* We thank the editor for centering the conversation around health literacy. To further explore the health literacy section added to the introduction (see above), we have added the sentences to the discussion as follows.

*“Additionally, patients are surrounded by easily digestible yet inaccurate medical information through social media and the internet which may be preferred to society endorsed PEMs[3, 17]. Therefore, an integration of AI with human editing and endorsement is the logical next step to enhance the dissemination and adoption of validated information about disease and treatment.”* (Page 13, lines 430-433)

The studies cited are as follows:

S. Loeb *et al.*, "Fake News: Spread of Misinformation about Urological Conditions on Social Media," (in eng), *Eur Urol Focus,* vol. 6, no. 3, pp. 437-439, May 15 2020, doi: 10.1016/j.euf.2019.11.011.

G. E. Cacciamani *et al.*, "Asking "Dr. Google" for a Second Opinion: The Devil Is in the Details," (in eng), *Eur Urol Focus,* vol. 7, no. 2, pp. 479-481, Mar 2021, doi: 10.1016/j.euf.2019.10.011.

**6) Need to include a limitation section that:**

- **discusses the potential limitations in the assessment methodology (small sample of expert reviewers, potential biases)**
- **discusses Likert scale's ability to detect meaningful differences when most ratings clustered around 4**
- **reflects on whether urologists are the ideal evaluators of patient-oriented materials**
- **any other limitations**

*Response:* We thank the editor for the comment and agree that the limitations section should refelct the pontential limitaitons listed above. We have encorporated those limitations into the text as follows:

*“Several limitations must be considered. First, the number of expert reviewers in the randomized assessment was limited due to the availability of YAU working group members. While steps were taken to minimize the potential bias of reviewers using a single-blinded, randomized assessment, bias can still be present given the distinct syntactic and lexical hallmarks found with GenAI output[43, 44]. Further, while Likert scales are often used in the evaluation of GenAI-generated content[11, 13], the ability to detect differences is often limited given the clustering of responses. Validated tools for evaluating the quality of GenAI-generated content are needed. Moreover, GenAI technology evolves quickly - while initially guidelines were input as text chunks, they can now be input as entire websites of PDF files directly and therefore integrated seamlessly into GPTs. Given this, the reproducibility of these findings is difficult to predict over longer timespans, however GenAI tools should regularly be reassessed to insure similar or improved performance. Finally, the understandability of the content has not been tested in patients. However, readability scores serve as an established tool with defined thresholds[5]. Additionally, the Bridging Readable and Informative Dissemination with GenerativE Artificial Intelligence (BRIDGE-AI) Initiative (OSF:* [*https://osf.io/8yz6d/*](https://osf.io/8yz6d/)*) is actively working with patient advocacy groups representing major genitourinary cancers to conduct the first randomized controlled trial evaluating how well urology patients understand GenAI-generated content.”* (Pages 13-14, lines 434-450)

Studies cited:

P. Reviriego, J. Conde, E. Merino-Gómez, G. Martínez, and J. A. Hernández, "Playing with words: Comparing the vocabulary and lexical diversity of ChatGPT and humans," Machine Learning with Applications, vol. 18, p. 100602, 2024/12/01/ 2024, doi: https://doi.org/10.1016/j.mlwa.2024.100602.

H. Rafique, I. Nazeer, and J. Rehman, "The Impact of ChatGPT on Language Evolution: A Linguistic Analysis," Journal of Education and Social Studies, vol. 5, no. 1, pp. 56-68, 02/23 2024, doi: 10.52223/jess.2024.5106.

M. B. Eppler et al., "Bridging the Gap Between Urological Research and Patient Understanding: The Role of Large Language Models in Automated Generation of Layperson's Summaries," (in eng), Urol Pract, vol. 10, no. 5, pp. 436-443, Sep 2023, doi: 10.1097/upj.0000000000000428.

Y. B. Shah, A. Ghosh, A. Hochberg, J. R. Mark, C. D. Lallas, and M. S. Shah, "Artificial intelligence improves urologic oncology patient education and counseling," (in eng), Can J Urol, vol. 31, no. 5, pp. 12013-12018, Oct 2024.

B. Weis, "Health Literacy: A Manual for Clinicians. Chicago, IL: American Medical Association, American Medical Foundation; 2003.• National Institutes of Health. How to Write Easy to Read Health Materials: National Library of Medicine Website," How to Write Easy to Read Health Materials: National Library of Medicine Website.

**Reviewer #1 Comments**

**1) Abstract, Line 158: The sentences "A randomized assessment of the correctness of these materials was conducted by 32 urologists and urology residents." and "Thirty-two members of the Young academic urologists (YAU) groups evaluated the accuracy, completeness, and clarity of the original versus GPT-generated PEMs." seem to repeat the same information. Please use just one of them to avoid redundancy.**

*Response:* We thank the reviewer for the comment and agree that this portion of the abstract lacked clarity and was redundant. To remedy this, we have modified this section of the abstract to remove the first use of the redundant phrase as follows:

*“Additionally, the PEMs were translated into five commonly spoken languages within the European Union (EU). The study was conducted through a single-blinded, online randomized assessment survey. After an initial pilot assessment of the GenAI-generated PEMs, thirty-two members of the Young Academic Urologists (YAU) groups evaluated the accuracy, completeness, and clarity of the original versus GPT-generated PEMs.”* (Page 3, lines 104-108)

**2) This same comment applies to the same section for the sentences "Additionally, translations into other languages were evaluated for correctness and clarity by native-speaker urologists." and "The translation assessment involved two native speakers from different YAU groups for each language: Dutch, French, German, Italian, and Spanish."**

*Response:* We thank the reviewer for the comment and agree that this is redundant, we have corrected this by modifying the first few sentences as follows:

*“Additionally, the PEMs were translated into five commonly spoken languages within the European Union (EU). The study was conducted through a single-blinded, online randomized assessment survey. After an initial pilot assessment of the GenAI-generated PEMs, thirty-two members of the Young Academic Urologists (YAU) groups evaluated the accuracy, completeness, and clarity of the original versus GPT-generated PEMs.* *The translation assessment involved two native speakers from different YAU groups for each language: Dutch, French, German, Italian, and Spanish.”* (Page 3, lines 104-109)

**3)** **Abstract, results: Please mention the number of original PEMs and AI generated PEMs assessed here, not just in the main results section.**

*Response:* The reviewer points out an important piece of information to include in the abstract; the number of PEMs (8) in each arm are now reported as follows:

*“The readability scores for the 8 original PEMs were lower than for the 8 GPT-4-generated PEMs (Mean FKRE: 43.5 vs. 70.8; p<.001).”* (Page 3, lines 116-117)

**4) The fact that translations of GPT-generated PEMs were rated as correct in 77.5% of cases and clear in 67.5% of cases, which indicates an error rate that should not be neglected and that AI is still not very reliable to translate such information without human supervision to avoid misinformation and chaos. Please elaborate on this in the discussion and highlight as a point of weakness in these generated PEMs, aside from highlighting their strength in more proper readability when compared to original PEM.**

*Response:* We thank the reviewer for the comment highlighting the need to discuss the weaknesses and inconsistency of PEM translation by GenAI models. We agree that human supervision is absolutely required in the case of translation by expert translators and bilingual physicians to ensure faithfulness and clarity. We have refined the discussion section to highlight the importance of human oversight in this case as follows:

*“Translations of GenAI-generated PEMs were rated as clear and faithful in more than two-thirds of cases, enabling the potential dissemination within the member states of the EAU after further refinement.”* (Page 11, lines 364-366)

*“Resolving the limitations may be possible through integration of GenAI into the workflow of updating PEMs. Integration with human verification and editing might allow organizations to keep up with the workload of guideline changes and increase readability while also maintaining the quality (Fig 1). This same workflow extends to translating PEMs. While the language capabilities will likely improve with future models[33], native-speaking urologists and certified translators should always work to finalize the translations prior to widespread dissemination.”* (Page 12, lines 402-407)

**5) FKRE should stand for Flesch Kincaid Reading Ease, not Flesch Reading Ease. Please correct it in the entire manuscript.**

*Response:* We thank the reviewer for pointing out this error and we have corrected it throughout the manuscript.

**6) Results, Line 368: The readability assessment is represented in figure 2, and not figure 1 as the text claims. Please correct it. Also, in line 388. The correctness assessment is represented in figure 3, and not figure 2 as the text says. The same error is in line 395 (Should be figure 4 instead of 3).**

*Response:* We thank the reviewer for the comment and have corrected the figure citations in line with PLOS guidelines throughout the manuscript.

**7) There is no Supplementary Table 3, and there is a jump from Supp. Table 2 to 4. Please rename 4 to 3 and correct in the text as well.**

*Response:* We thank the reviewer for the comment and have corrected the supplementary figure/table naming in line with PLOS guidelines throughout the manuscript.
